# Cannabinomimetric Lipids: From Natural Extract to Artificial Synthesis

**DOI:** 10.1007/s13659-017-0151-9

**Published:** 2018-01-16

**Authors:** Ya-Ru Gao, Yong-Qiang Wang

**Affiliations:** Key Laboratory of Synthetic and Natural Functional Molecule Chemistry of Ministry of Education, Department of Chemistry & Materials Science, Northwest University, Xi’an, 710069 People’s Republic of China

**Keywords:** Endocannabinoid, Cannabinoid receptors, Cannabinomimetric lipids

## Abstract

Endocannabinoid system is related with various physiological and cognitive processes including fertility, pregnancy, during pre- and postnatal development, pain-sensation, mood, appetite, and memory. In the latest decades, an important milestone concerning the endocannabinoid system was the discovery of the existence of the cannabinoid receptors CB_1_ and CB_2_. Anandamide was the first reported endogenous metabolite, which adjusted the release of some neurotransmitters through binding to the CB_1_ or CB_2_ receptors. Then a series of cannabinomimetric lipids were extracted from marine organisms, which possessed similar structure with anandamide. This review will provide a short account about cannabinomimetric lipids for their extraction and synthesis.

## Introduction

**Fig. 1:**
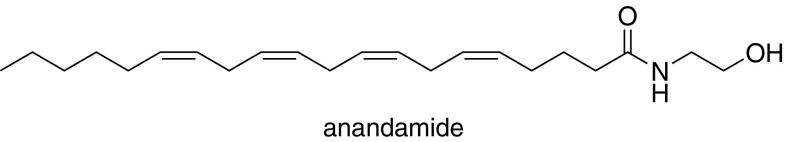
The structure of anandamide

In the past decades, pharmacologists devoted more interest to the study of endocannabinoid system due to its relation with various physiological and cognitive processes including fertility, pregnancy, during pre- and postnatal development, pain-sensation, mood, appetite, and memory [1]. Originally, the endocannabinoid system was discovered while scientists tried to understand the physical and psychological effects of cannabis, thereby named it the endocannabinoid system for this reason. An important milestone concerning the endocannabinoid system was the discovery of the existence of the cannabinoid receptors (CB_1_ and CB_2_) in central and peripheral mammalian tissues [2–5]. Both receptors CB_1_ and CB_2_ belong to the large family of G-protein-coupled receptors (GPCR). CB_1_ receptor exhibits a widespread distribution in the mammalian brain and are responsible for the psychological and anticonvulsive effects produced by marijuana [2–5], while CB_2_ receptor is most abundant in the immune and hematopoietic system and is involved in the anti-inflammatory and possibly other therapeutic effects of cannabis [4, 5]. The discovery of cannabinoid receptors (CB_1_ and CB_2_) has launched the quest for endogenous ligands of these receptors. Based on the assumption that the endogenous cannabinoid ligand was a lipid soluble compound, a lipid derivative was first isolated from chloroform–methanol extracts of porcine brain and christened anandamide by Mechoulam et al. in 1992 (Fig. 1) [6]. This endogenous metabolite bound to both CB_1_ and CB_2_ receptors and was found in nearly all tissues in a wide range of animals [7]. Then a series of alkyl amides were extracted from marine organisms, which resembled structurally some aspects of anandamide and had been termed cannabimimetic lipids. In the biological activity tests, they showed the ability to bind and activate at least one cannabinoid receptor [8]. This review will provide a short account of cannabinomimetric lipids for their natural extract and artificial synthesis.

## Extraction and Biological Activities (Fig. 2)

### Grenadamide

Grenadamide was isolated from the organic extract of a Grenada collection of the marine cyanobacterium *Lyngbya majuscula* by Gerwick et al. in 1998. It exhibited brine shrimp toxicity (LD_50_ = 5 μg/mL) and modest cannabinoid receptor binding activity (*K*_*i*_ = 4.7 μM) [9]. Gerwick et al. verified the structure and the relative stereochemistry of grenadamide, which was a *trans*-cyclopropyl-containing fatty acid-derived metabolite.

**Fig. 2:**
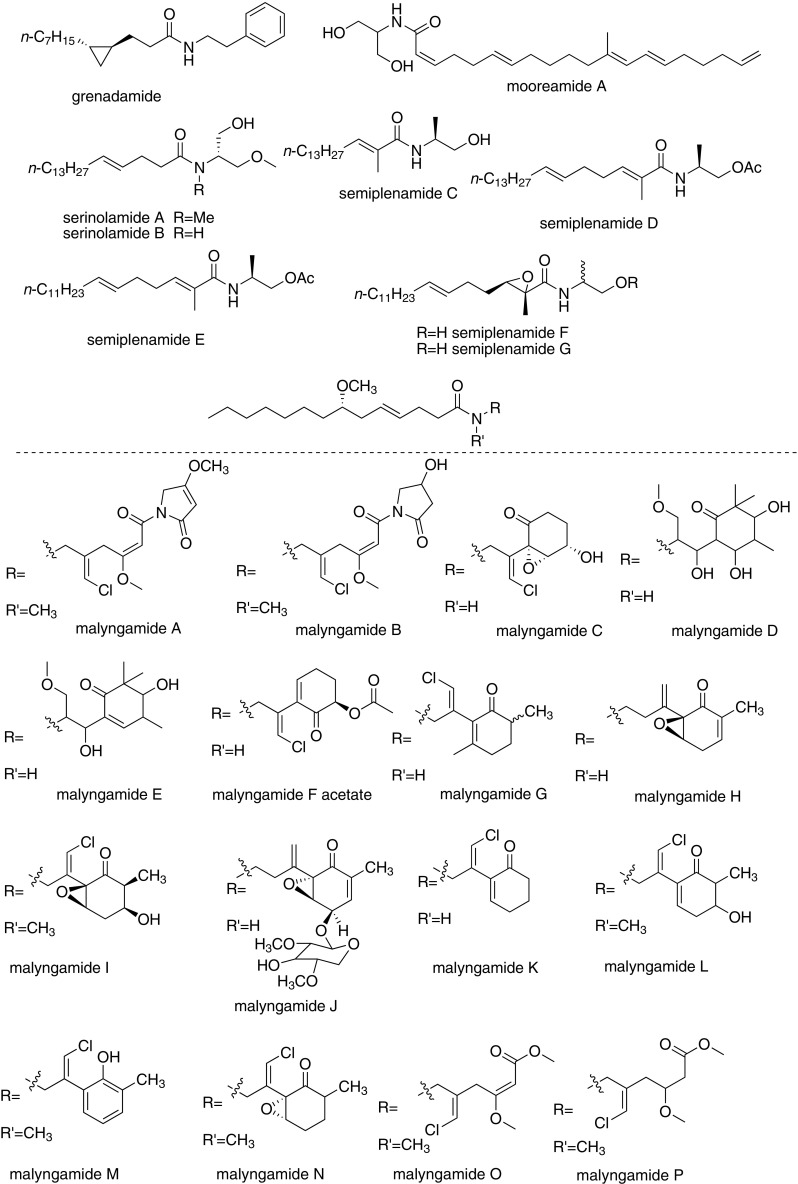

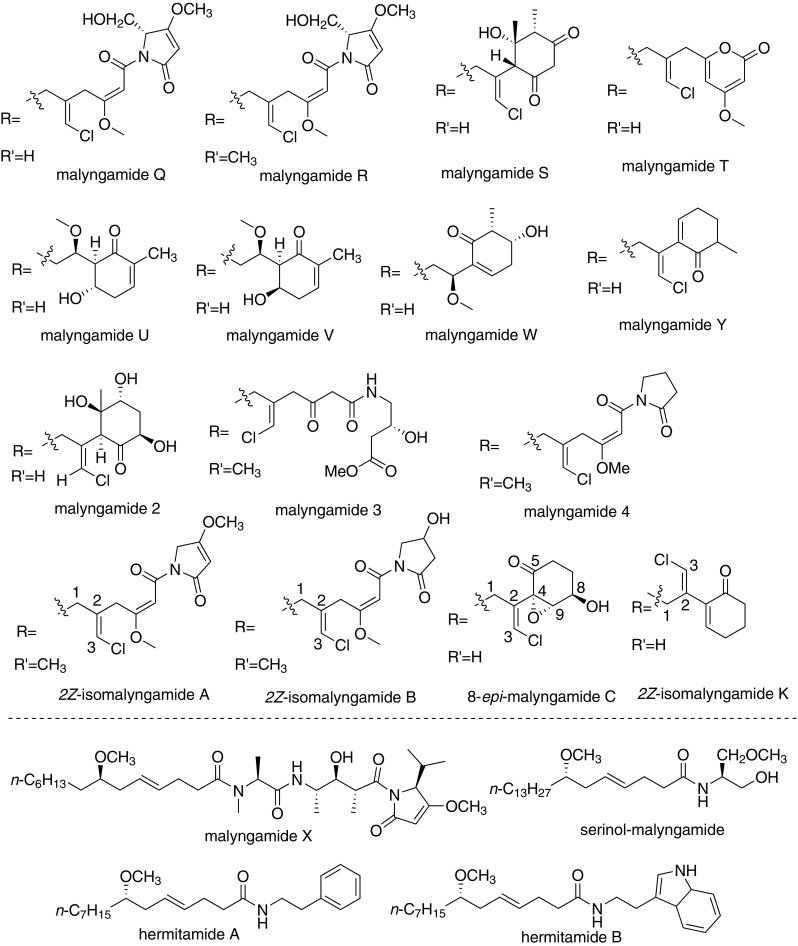
The structures of cannabinomimetric lipids

### Mooreamide A

Mooreamide A was extracted from cyanobacterium *Moorea bouillonii* by Gerwick et al. from Papua New Guinea, and showed strong and selective affinity to CB_1_ ligand [10].

### Serinolamides

Serinolamide A was isolated from marine cyanobacteria *Lyngbya majuscule* collected in Papua New Guineal. It displayed a moderate agonist effect and selectivity for the CB_1_ cannabinoid receptor [11]. Serinolamide B, a closely related analogue of serinolamide A, was isolated from a Lyngbya sample from the piti Bomb Holes in Guam by Luesch et al. [12]. It showed moderate affinities to both CB_1_ and CB_2_, while exhibited a higher selectivity for CB_2_ (*K*_*i*_ = 5.2 μm) over CB_1_ (*K*_*i*_ = 16.4 μm)

### Semiplenamides

Semiplenamides A to G were isolated from the marine cyanobacterium *Lyngbya semiplena* collected from Papua New Guinea by Gerwick et al. [13]. In the test of their affinity to cannabinoid receptors of the rat brain membranes, only semiplenamides A, B and G worked. In the test of fatty acid amide hydrolase (FAAH), the semiplenamides A to G were not found appreciable inhibitory effect.

### Malyngamides

Malyngamides include over 30 members, characterized by different *N*-substitution groups of amides. They were isolated from Marine cyanobacterium *Lyngbya majuscule*, and showed a wide range of biological activities, such as antifeedant activity, ichthyotoxicity, toxicity to other marine animals, cytotoxicity to cancer cells, anti-HIV, anti-leukemic, and anti-tumor activity [14–16]. The finding that malyngamide B possesses cannabimimetic properties provides new insight into the biological activities of malyngamides. The extraction information of malyngamides was illustrated in Table 1.

**Table 1:** The extraction of Malyngamides

Malyngamides	Source	Reference
Malyngamide A	Marine cyanophyte *Lyngbya majuscula*	Cardellina II et al. [20]
Malyngamide B	Blue-green alga *Lyngbya majuscule*	Cardellina II et al. [21]
Malyngamide C	Shallow-water variety of *Lyngbya majuscula* found on the reefs of Fanning Island in the Line Islands	Ainslie et al. [22]
Malyngamides D, E	Deep water variety of the marine cyanophyte *Lyngbya majuscula*	Mynderse et al. [23, 24]
Malyngamides F	Cyanobacterium *Lyngbya majuscula*	Villa et al. [25]
Malyngamide G	Blue-green alga epiphyte of the brown mediterranean alga Cystoseira crinita	Praud et al. [26]
Malyngamide H	Tropical marine cyanobacteriurn *Lyngbya mjuscula*	Orjala et al. [27]
Malyngamide I	Tropical marine cyanobacterium *Lyngbya* *majuscula*	Todd et al. [28]
Malynsamides J, K, L	Marine cyanobaeterium *Lyngbya majuscula*	Wu et al. [29]
Malyngamide M, N	Hawaiian red alga *Gracilaria coronopifolia*	Kan et al. [30]
Malyngamides O, P	Sea hare *Stylocheilus longicauda*	Gallimore et al. [31]
Malyngamides Q, R	Madagascan *Lyngbya majuscula*	Milligan et al. [32]
Malyngamides S	New Zealand collection of the sea hare *Bursatella leachii*	Appleton et al. [33]
Malyngamides T	Puerto Rican collection of *Lyngbya majuscula*	Nogle et al. [34]
Malyngamides U, V, W	Marine cyanobacterium *Lyngbya majuscula* collected in Papua New Guinea	McPhail et al. [35]
Malyngamide X	Thai sea hare *Bursatella leachii*	Suntornchashwej et al. [36]
Malyngamide Y	a Florida collection of *Moorea producens*	Sabry et al. [37]
Malyngamide 2	marine cyanobacterium cf. *Lyngbya sordida*	Malloy et al. [38]
Malyngamide 3	*Lyngbya majuscula* from Cocos Lagoon, Guam	Gunasekera et al. [39]
Malyngamide 4	Red Sea marine cyanobacterium *Moorea producens*	Shaala et al. [40]
Isomalyngamides A, B	cyanobacterium *Lyngbya majuscula* from Hawaiian waters	Kan et al. [41]
8-Epi-malyngamide C	Floridian marine cyanobacterium *Lyngbya majuscula*	Kwan et al. [42, 43]
2Z-isomalyngamide K	Papua New Guinea field collection of the cyanobacterium *Lyngbya majuscula*	Han et al. [44]
Serinol-derived malyngamide	Australian Cyanobacterium	Wan et al. [45]

Hermitamides resemble the malyngamide-type compound in structure and were still isolated from the marine marine cyanobacterium *L. majuscula* of other species of Gracilaria [17–19]. Hermitamides were evaluated for their biological activity in several systems. Hermitamides A (1) and B (2) showed LD_50_ values of 5 μM and 18 μM respectively in the brine shrimp (Artemia salina) toxicity assay, and showed IC_50_ values of 2.2 μM and 5.5 μM respectively to Neuro-2a neuroblastoma cells in tissue culture.

## Synthesis of Cannabinomimetric Lipids

### Synthesis of Grenadamide

**Scheme 1:**
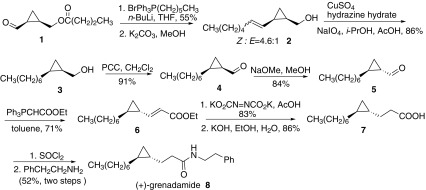
Synthesis of grenadamide reported by Baird [9]

In 2004, Baird and co-workers reported the synthesis of grenadamide and confirmed its absolute stereochemistry (Scheme 1) [9]. The synthesis started from the aldehyde **1**, which was converted to olefin **2** through Wittig reaction the following ester hydrolysis. Then removal of the double bond gave **3**. Oxidation of alcohol **3** got aldehyde **4** and epimerisation of **4** using sodium methoxide in methanol afforded the epimer **5**. Then **5** underwent HWE reaction with ethoxycarbonyl triphenylphosphosphorane to give the ester **6**, which was removed the double bond with di-potassium azodicarboxylate and hydrolysed with KOH to afford acid **7**. The compound **7** was converted into the corresponding chloride, then treated with 2-phenylethylamine to give the amide **8**, which had an equal and opposite absolute rotation compared with natural grenadamide. So the synthetic sample was the enantiomer of the natural product grenadamide.

**Scheme 2:**
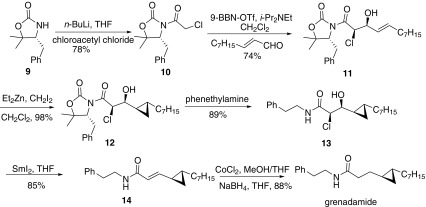
Synthesis of grenadamide reported by Bull [46]

One year later, Bull and co-workers reported an asymmetric synthesis of grenadamide in 6 steps using (*R*)-5,5-dimethyl-oxa-zolidin-2-one as a chiral auxiliary (Scheme 2) [46]. The starting material **9** as a chiral auxiliary was acetylated with chloroacetyl chloride to give **10**. Then treatment of **10** with 9-BBN–OTf and *i*-Pr_2_NEt and following reaction with *α*,*β*-unsaturated aldehyde afforded *syn*-aldol product **11** in 92% de. Cyclopropanation of **11** with Et_2_Zn and CH_2_I_2_ afforded **12** with high stereoselectivity. Then replacing the oxazolidin-2-one fragment with phenylethylamine gave **13**, which was treated with SmI_2_ resulted in clean elimination reactions to afford (*E*)-*α*,*β*-unsaturated amide. Finally reduction of the double bond with NaBH_4_ and CoCl_2_ afford grenadamide.

**Scheme 3:**
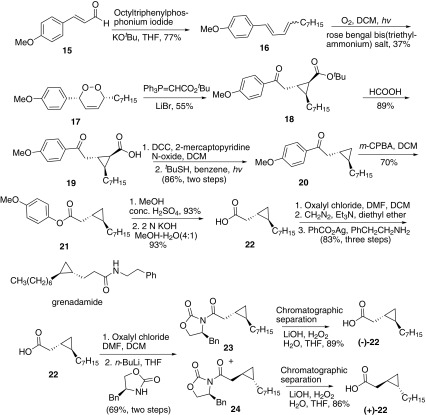
Synthesis of grenadamide reported by Taylor [47]

In the same year, Taylor and co-workers reported the total synthesis of both (+)-grenadamide and (−)-grenadamide by a racemic route (Scheme 3) [47]. Wittig reaction of 4-methoxycinnamaldehyde **15** and octyltriphenylphosphonium iodide resulted the 1,3-diene **16**. Then photolysis of 1,3-diene **16** gave the 1,2-dioxine **17**, which reacted with *tert*-butyl ester ylide to afford cyclopropane **18**. Then hydrolysis of *tert*-butyl ester group with formic acid gave acid **19**, which was subsequently decarboxylated using the Barton protocol to afford **20**. Baeyer–Villiger oxidation of **20** using *m*-CPBA proceeded with excellent selectivity to give phenol ester **21**, which was subsequently hydrolyzed to give cyclopropyl fatty acid **22**. Arndt-Eistert homologation/amidation of acid **22** afforded racemic grenadamide. To the further study, the author obtained the two enantiomers of grenadamide. Fatty acid **22** was coupled with Evans’ auxiliary and chromatographically separating the diastereomers **23** and **24**, which were removed of the auxiliary to give (−)-**22** and (+)-**22** respectively. Then the enantiomerically pure fatty acids were subjected to the Arndt-Eistert protocol to give (−)-grenadamide and (+)-grenadamide.

**Scheme 4:**
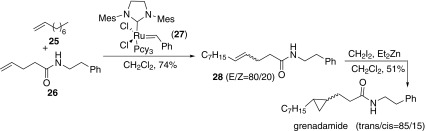
Synthesis of grenadamide reported by Piva [48]

In 2007, Piva and co-workers reported the synthesis of racemic grenadamide through a sequential cross-metathesis/Simmons–Smith cyclopropanation (Scheme 4) [48]. Cross-metathesis of **26** with 1-nonene **25** catalyzed by Grubbs type catalyst **27** delivered **28** as mixture of E and Z isomers. Then cyclopropanation of the mixture of **28** afforded grenadamide.

**Scheme 5:**
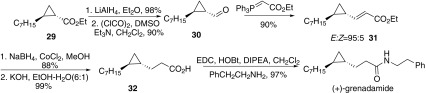
Synthesis of grenadamide reported by Boysen [49]

In 2010, Boysen and co-workers reported an asymmetric synthesis of (+)-grenadamide, an enantiomer of the natural product (−)-grenadamide (Scheme 5) [49]. The cyclopropyl carboxylic ester **29** was transformed into the corresponding aldehyde **30** by reduction with lithium aluminium hydride to alcohol, followed by Swern oxidation. Then aldehyde **30** underwent Wittig olefination to give *α*,*β*-unsaturated ester **31**. Reduction of **31** and followed by hydrolysis afforded acid **32**, which was coupled with phenethylamine gave (+)-grenadamide.

### The Synthesis of Serinolamide A

**Scheme 6:**
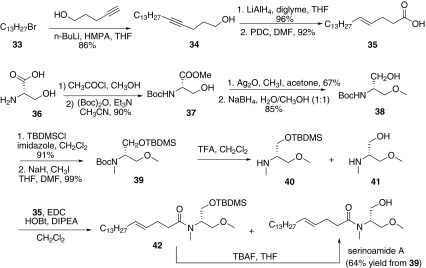
Synthesis of (+)-serinolamide A reported by Wang [50]

In 2013, our group reported the first total synthesis of (+)-serinolamide A in nine steps from l-serine with 30% overall yield (Scheme 6) [50]. The synthesis of (+)-serinolamide A was divided into two parts, the fatty acid **35** and the derivatives of serinol **40** and **41**. The coupling of 1-bromotridecane and pent-4-yn-1-ol using excess *n*-BuLi in HMPA afforded **34**. Then reduction of **34** with LiAlH_4_ afforded alkene and following oxidation of hydroxyl with PDC gave fatty acid **35**. The other key part was synthesized from l-serine. Esterification of **36**, followed by protection of the amino group afforded **37**. Methylation of **37** with iodomethane and then reduction of ether group with NaBH_4_ gave **38**. Protection of the hydroxyl group with TBDMSCl, subsequent *N*-methyl using MeI and NaH afforded **39**. Deprotection of Boc group gave the mixture of **40** and **41**, which without purification and separation condensed with **35** to afford the corresponding products **42** and serinolamide A. Then **42** was treated with TBAF to convert into serinoamide A as well.

**Scheme 7:**
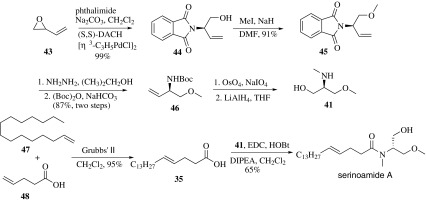
Synthesis of (+)-serinolamide A reported by Pandey [51]

Later, Pandey and co-workers reported the synthesis of (+)-serinolamide A in 2015 from the starting material of butadiene monoepoxide in five steps with 51% yield (Scheme 7) [51]. The compound **43** under the Trost’s DyKAT conditions reacted with phthalimide furnished asymmetric allylic alkylation derivative pthaloyl alcohol **44** in high regio- and enantioselective. Etherification of **44** with MeI in presence of NaH furnished **45**. Cleavage of phthalimide with hydrazine and then protection of the primary amine with (Boc)_2_O gave **46**. Then oxidative cleavage of terminal double bond gave the aldehyde, which was under reduction conditions to afford the serino derivative **41**. The other part fatty acid **3** was synthesized from pentadec-1-ene **47** and 4-pentenoic acid **48** undertaken RCM reaction. The fatty acid **35** was then condensed with serino derivative **41** to afford serinoamide A.

### Synthesis of Semiplenamides

**Scheme 8:**
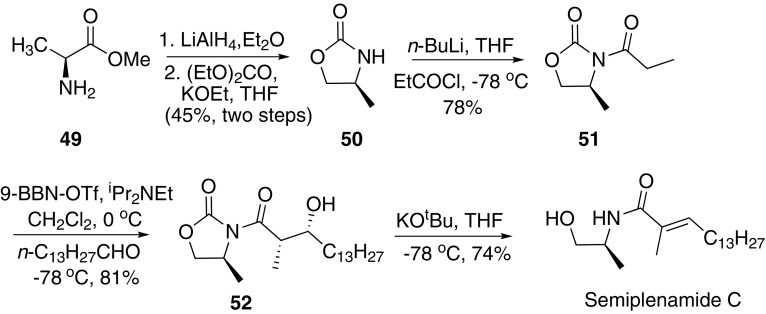
Synthesis of semiplenamide C reported by Bull [52]

In 2005, Bull and co-workers developed an efficient method for the synthesis of (*E*)-*α*,*β*-unsaturated amide and applied the methodology for the synthesis of semiplenamide C (Scheme 8) [52]. l-Alanine methyl ester **49** was chosen as the starting material. Reduction of **49** with LiAlH_4_ and then protection with diethyl carbonate afford **50**. Subsequent treatment of **50** with *n*-BuLi and propionyl chloride gave **51**. Then pretreatment of **51** with 9-BBN-OTf and *i*-Pr_2_NEt underwent an aldol reaction with tetradecanal to give **52** in > 95% de. Finally, deprotection of **52** with KO^*t*^Bu afforded semiplenamide C.

**Scheme 9:**
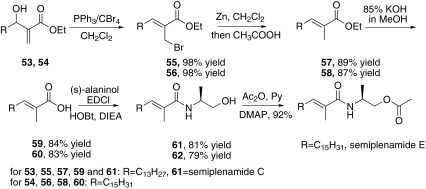
Synthesis of semiplenamide C reported by Das [53]

In 2009, Das and co-workers reported the synthesis of semiplenamides C and E through the Baylis–Hillman adducts [53]. (Scheme 9) The Baylis–Hillman adducts **53** and **54** were treated with PPh_3_/CBr_4_ afforded the corresponding allyl bromides **55** and **56**, which were subsequently treated with Zn and CH_3_COOH to give **57**, **58** respectively. The esters **57** and **58** were then hydrolyzed with KOH to give the corresponding acids **59** and **60**, which were condensed with (*S*)-alaninol to form semiplenamide C (**61**) and **62** respectively. Compound **62** was further acetylated with acetic anhydride to furnish semiplenamide E.

### Synthesis of Malyngamides

**Scheme 10:**
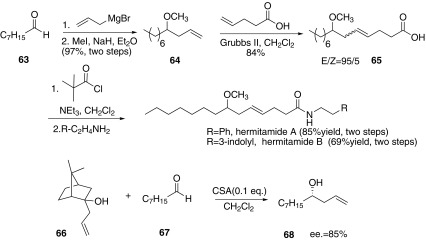
Synthesis of hermitamides A and B reported by Piva [54]

In 2006, Piva and co-workers reported both the racemic synthesis and a formal enantioselective synthesis of hermitamides A and B (Scheme 10) [54]. Homoallylic ether **64** was prepared through the Grignard reaction from the octanal **63** and allyl magnesium bromide and the following protection of the hydroxyl group by MeI in the presence of NaH. Then **64** and butenoic acid proceeded RCM reaction to afford **65** with the E/Z ratio of 95/5. Finally, racemic hermitamide A and B were synthesized through the condensation of **65** with the phenethylamine and 3-indolyl-ethylamine respectively. For the further study, the author developed an enantioselective methodology for the synthesis of **68**, which was obtained through the reaction of (+)-camphor homoallylic alcohol **66** and octanal **67** catalyzed by CAS with the *ee* value of 85%.

**Scheme 11:**
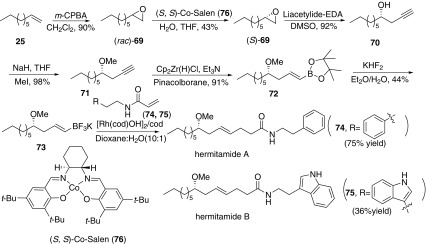
Synthesis of hermitamides A and B reported by Frost [55]

In 2009, Frost and co-workers reported an enantioselective synthesis of hermitamides A and B from the starting material 1-nonene **25** (Scheme 11) **[**55]. Epoxidation of **25** by *m*-CPBA afforded the racemic epoxide **69**. Then catalyzed by cobalt (II) complex **76**, *rac*-**69** underwent a hydrolytic kinetic resolution process to afford the chiral epoxide (*S*)-**69** in 96% *ee*. Subsequently, homopropargylic alcohol **70** was obtained when the addition of lithium acetylide complexed with EDA. After methylated the hydroxyl group, the compound **71** was converted to the chiral alkenylpinacol boronic ester **72** with the addition of catalytic Schwartz reagent and anhydrous triethylamine. However, **72** was failed to react with both phenylethylamine and tryptamine acrylamide under a range of conditions. Thus pinacolboronic ester **72** was converted to chiral alkenyltrifluoroborate salt **73**. Finally, alkenyltrifluoroborate salt **73** was reacted with phenylethylacrylamide **74** or **75** in the presence of [Rh(cod)(OH)]_2_ and cyclooctadiene as the ligand to afford hermitamides A and B respectively.

**Scheme 12:**
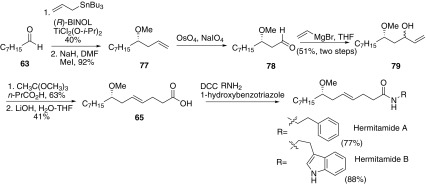
Synthesis of hermitamides A and B reported by Paige [56]

In 2011, Paige and co-workers reported an asymmetric synthesis of hermitamides A and B (Scheme 12) [56]. Asymmetric allylation of octanal with allyltributylstannane mediated by a titanium-binol complex gave homoallylic alcohol followed by the methylation with MeI to afford ether **77**. Oxidative cleavage of the terminal double bond yielded aldehyde **78**, which was reacted with vinylmagnesiun bromide to afford allylic alcohol **79**. The compound **79** was then generated Johnson-Claisen rearrangement with the addition of trimethylorthoacetate in the presence of catalytic amount of propionic acid to afford methyl ester of lyngbic acid followed by the saponification with lithium hydroxide to give **65**. Then acid **65** was coupled with phenethylamine or tryptamine to afford hermitamides A and B respectively.

**Scheme 13:**
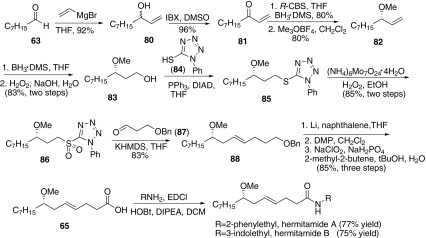
Synthesis of hermitamides A and B reported by Narender [57]

In 2014, Narender and co-workers reported a concise total synthesis of hermitamides A and B in a high enantioselectivity (Scheme 13) [57]. The synthesis was commenced from octanal **63**. Vinylation with vinyl magnesium bromide gave allylic alcohol **80**, which was oxidated by IBX to afford enone **81**. Then asymmetric reduction of ketone **81** catalyzed by CBS gave the chiral allylic alcohol with high enantioselectivity, which was treated with Meerwein’s reagent to afford the methylation product (*S*)-**82.** Subsequently, hydroboration-oxidation of **82** afforded the primary alcohol **83**. Then **83** with 1-phenyltetrazole-5-thiol (**84**) underwent Mitsunobu reaction to give aryl sulfide **85**, which was further oxidated by ammonium molybdate and hydrogen peroxide to give sulfone **86**. Coupling of alkyl sulfone **86** with aldehyde **87** via Julia-Lythgoe olefin provided the corresponding olefin **88** with *E*-geometry exclusively. Then removal of benzyl obtained the primary alcohol following oxidation of the hydroxyl group to furnish lyngbic acid **65**. Acid **65** was coupled with 2-phenylethyl amine or 3-indolylethylamine to provide hermitamides A and B, respectively.

**Scheme 14:**
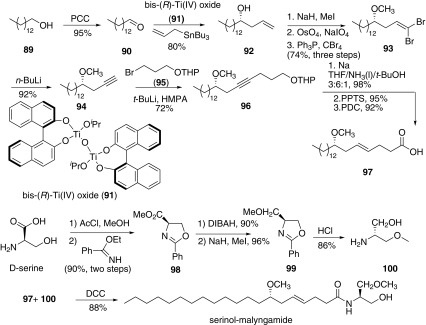
Synthesis of serinol-malyngamide reported by Cao [58]

In 2006, Cao and co-workers reported the synthesis of serinol-malyngamide for the first time (Scheme 14) [58]. The molecular was divided into two parts, the fatty acid **97** and the derivative of serinol **100**. The stereoselective synthesis of fatty acid **97** had been reported previous [59], which was starting from 1-tetradecanol. Oxidation of **89** afforded the aldehyde **90**, which further reacted with allyltributyltin catalyzed by bis-(*R*)-Ti(IV) oxide (**91**) to afford allyl alcohol **92**. Methylation of hydroxyl group and followed by oxidation of terminal olefin afforded aldehyde, subsequently reacted with PPh_3_/CBr_4_ to produce dibromide **93**. Then alkyne **94** was obtained after the addition of *n*-BuLi. Alkyne coupled with **95** gave **96**, reduction of which obtained olefin with *E*-configuration. Sequent deprotection of THP group and oxidation of the obtained primary alcohol afforded fatty acid part **97**. The other part of the derivative of serinol **100** would be synthesized from chiral starting material d-serine by the method of Meyers et al. Finally the acid **97** and the derivative of serinol **100** were condensed under the DCC conditions to afford serinol-malyngamide.

**Scheme 15:**
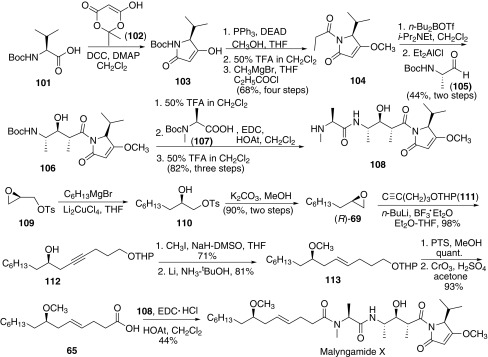
Synthesis of malyngamide X reported by Isobe [60]

In 2007, Isobe and co-workers reported the synthesis of malyngamide X [60], which possessed an unusual tripeptide portion connecting to a methoxylated fatty acyl chain (Scheme 15). Malyngamide X still was divided into two parts tripeptide portion **108** and fatty acid portion **65**. The synthesis of tripeptide portion was started from commercially available *N*-Boc-l-valine (**101**), which was coupled with Meldrum’s acid (**102**) to afford pyrrolidone derivative **103**. Then sequent Mitsunobu reaction, deprotection of Boc group and following *N*-propionylation occurred, *N*-propionyl pyrrolidone **104** was obtained. Subsequently, **104** was then coupled with *N*-Boc-l-alaninal (**105**) assisted by *n*-BuBOTf to afford the pyrrolidone derivative **106**. Then the coupling of **106** and Boc-*N*-Me-l-alanine gave the desired tripeptide segment **108**. The other part was started from *R*-glycidyl tosylate (**109**). Thus, Grignard reaction of **109** with C_6_H_13_MgBr gave the desired alcohol **110**. Then conversion of alcohol **110** to epoxynonane (+)-**69** and the following coupling with acetylide **111** gave homopropargylic alcohol **112**. Methylation of alcohol and the subsequent reduction of acetylene provided **113** with *E*-geometry. Cleavage of THP protecting group and the following oxidation of hydroxyl group gave fatty acid **65**. Finally, coupling of lyngbic acid segment **65** with amine **108** gave malyngamide X.

**Scheme 16:**
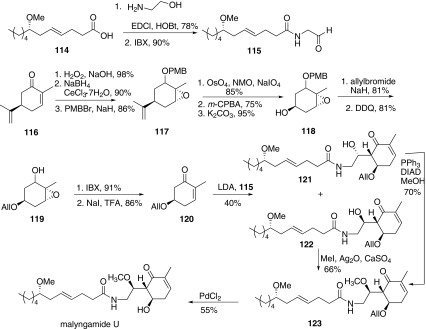
Synthesis of malyngamide U reported by Cao [62]

In 2006, Cao and co-workers reported the total synthesis malyngamide U [61] and revised its correct absolute configuration. The later year, they reported an improved asymmetric synthesis of malyngamide U [62] from the same material and similar methodology (Scheme 16). The fatty acid **114** was synthesized as the same way illustrated in Scheme 13, and the starting material was hexanal instead of tetradecanal. Then amidation of acid **114** with ethanolamine and oxidation of the obtained primary alcohol provided amido-aldehyde **115**. The other part started from (*R*)-(−)-carvone **116**, which was indicated to convert to **120** for the further aldol reaction with amido-aldehyde **115**. Epoxidation of (*R*)-(−)-carvone, reduction of ketone moiety under Luche’s conditions and following protection of the hydroxyl group with *p*-methoxybenzyl (PMB) generated **117**. Then oxidation of the terminal double bond to ketone and further Baeyer–Villiger rearrangement of ketone with *m*-CPBA gave the corresponding acetate. Removal of acetyl group afforded the secondary alcohol **118**. Subsequently the alcohol **118** was protected using allyl bromide and removal of the protecting group PMB with DDQ gave **119**. Then the oxidation of the resulted alcohol got ketone by IBX and the reduction of the epoxide on the work of Adams provide the key intermediate **120**. Aldol condensation of **120** with amido-aldehyde **115** afforded two epimers **121** and **122**. The configuration of **122** was in accordance with malyngamide U. Thus methylation of **122** with MeI gave **123**, which was also obtained by Mitsunobu reaction of **121**. Finally, removal of the allyl protecting group completed the synthesis of malyngamide U.

**Scheme 17:**
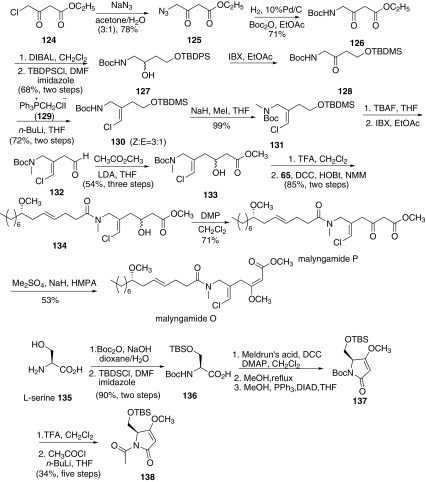

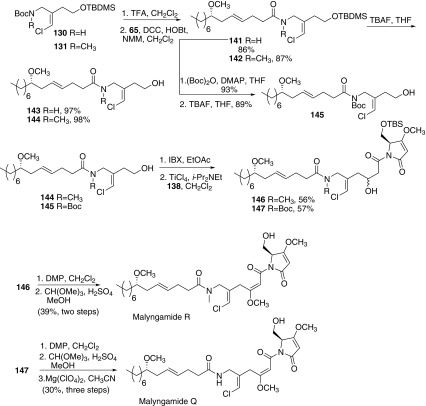
Synthesis of malyngamide O, P, Q and R reported by Cao [63]

In 2009, Cao and co-workers reported a convergent route for the total synthesis of malyngamides O, P, Q, and R (Scheme 17) [63]. Preparation of key intermediate **131** began with ethyl 4-chloro-3-oxobutanoate **124**. Azidation of **124** afforded azide **125**, which was subsequently hydrogenated by H_2_ in the presence of di-tert-butyl dicarbonate gave the Boc-protected amine **126**. Reduction of both keto and ester carbonyl groups in ester **126** with diisobutylaluminum hydride (DIBAL-H) afforded the corresponding diol, followed by monoprotection of the primary hydroxy group with *tert*-butyldiphenylsilyl chloride (TBDPSCl) to give the corresponding silyl ether **127**. Then oxidation of secondary alcohol **127** with 2-iodoxybenzoic acid (IBX) provided the corresponding ketone **128**, which was subjected directly to Wittig olefination with chloromethyltriphenylphosphonium iodide (**129**) to give the vinyl chloride as a mixture of *Z*- and *E*-isomers (Z:E = 3:1). The Z-configuration of the vinyl chloride was consistent with that in natural malyngamides O and P. Then *N*-methylation of **130** provided the key vinyl chloride **131**. Thus, deprotection of the TBDMS group of **131** with TBAF, followed by oxidation of alcohol with IBX afforded aldehyde **132**. Then aldehyde **132** reacted with the enolate derived from methyl acetate in THF to give **133**. Racemic alcohol **133** was immediately submitted to deprotection of the Boc group to generate the corresponding amine, which was directly condensed with the carboxylic acid **65** to afford amide **134**. Finally, oxidation of **134** with Dess-Martin periodinane gave malyngamide P. Deprotionation and the following methylation provided malyngamide O. Then the authors continued to synthesize malyngamides Q and R, bearing the more challenging structure. The acetamide **138** bearing the pyrrolidone ring was prepared from l-serine **135**. Protection of the amino group and hydroxy group in l-serine with Boc_2_O and TBSCl respectively provided acid **136**. Then condensation reaction of **136** with Meldrum’s acid, followed by treatment with MeOH furnished the pyrrodidone intermediate, which was subjected to a Mitsunobu reaction to give O-methyl pyrrolidone derivative **137**. Removal of the Boc group with TFA provided pure amine, which was further protected by acetyl chloride provided N-acetyl pyrrolidone (*S*)-**138**. However, aldol reaction products of **138** and **132** could be achieved, the further condensation reaction with acid **65** was failed with little desired compound. Thus another strategy was adopted that was amidation of **130** and **131** first, and then conducted the aldol reaction with (*S*)-**138** in the second step. Removal of the Boc groups in **130** and **131**, followed by amidation with acid **65** produced amides **141** and **142**, respectively. Then deprotection of the TBDMS groups in **141** and **142** with TBAF gave the corresponding alcohols **143** and **144** respectively. *N*-Protection with (Boc)_2_O affored **145**. Oxidation of alcohol **144** and **145**, followed by condensation precursor enolate of pyrrolidone **138** afforded a diastereomeric mixture of alcohols **146** and **147** respectively. Oxidation of **146** and the following methylation of the enol afforded Malyngamide R. Using the similar route for the preparation of malyngamide R, enol methylation and subsequent removal of the Boc group gave malyngamide Q.

**Scheme 18:**
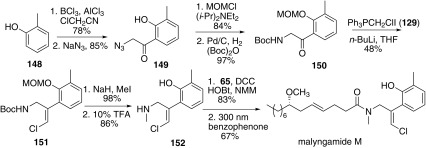
Synthesis of malyngamide M reported by Cao [64]

In 2010, Cao and co-workers reported the stereoselective synthesis of malyngamide M [64], which was still divided into two parts (Scheme 18). The lyngbic acid part **65** was achieved through the common methodology reported by their group (Scheme 13). The other amine part **152** possessing a vinylic chloride moiety was synthesized from *o*-cresol **148**. Friedel–Crafts acetylation of **148** with chloroacetonitrile and the following substitution reaction with sodium azide gave phenol **149**. Then protection of phenol with methoxymethyl chloride and subsequent hydrogenation of azide group by 10% Pd/C in situ protection with di-tert-butyl dicarbonate afforded **150**. Then **150** underwent Wittig olefination with chloromethyl triphenylphosphonium iodide to provided **151** with *Z* geometry. The amine part **152** was obtained through the sequent *N*-methylation and the simultaneous removal of both the MOM and Boc groups. Finally the coupling of acid part **65** and amine part **152** only afforded the isomalyngamide M with the Z-vinyl chloride in the structure, which was exposed to UV-light (λ > 300 nm) in the presence of benzophenone to afford malyngamide M (E-vinyl chloride).

**Scheme 19:**
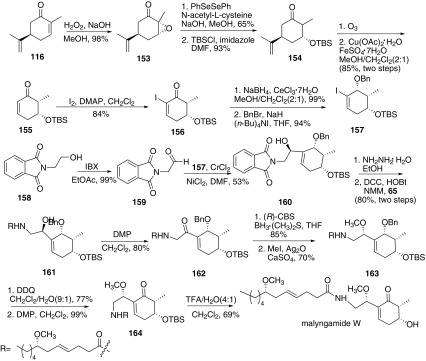
Synthesis of malyngamide W reported by Cao [65]

In 2011, Cao and co-workers reported the total synthesis of malyngamide W and confirmed the absolute configuration of malyngamide W (Scheme 19) [65]. *α*,*β*-Unsaturated cyclohexanone **155** was a key intermediate towards the synthesis, which was synthesized from (*R*)-(−)-carvone **116**. Epoxidation of **116** using hydrogen peroxide provided epoxy ketone **153**. Then the epoxide was opened and led to the 3a-hydroxyl ketone. Subsequently, the hydroxy group was further protected by *tert*-butyldimethylsilyl chloride to provide its TBS-ether **154**. Ozonolysis of **154** and subsequent treatment with copper (II) acetate monohydrate/iron sulfate heptahydrate furnished the enone **155**. Iodination of **155** obtained *α*-indo-*α*,*β*-unsaturated cyclohexone **156**, which underwent Luche’s reduction and subsequent protected by benzyl bromide to afford the key iodide **157**. The other part was synthesized from **158**, which was oxidized by IBX to provide the aldehyde **159**. Then **157** and **159** underwent Nozaki–Hiyama–Kishi coupling reaction to afford **160**, which was removed the *N*-protection group and coupled with lyngbic acid **65** to afford amide **161**. Oxidation of **161** with Dess–Martin periodinane afforded **162**. Reduction of **162** by Corey-Bakshi-Shibata oxazaborolidine (CBS catalyst) and methylation of the hydroxyl group afforded **163**. Then removal of the O-Bn protecting group with DDQ and subsequent oxidation of the alcohol with DMP afford amide **164**. Finally, removal of the TBS moiety gave malyngamide W.

**Scheme 20:**
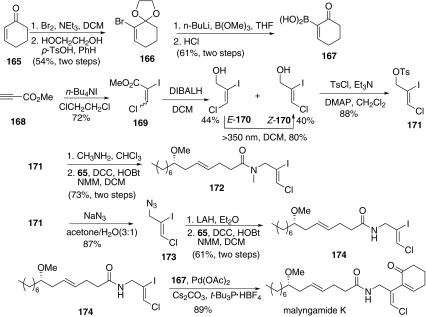
Synthesis of malyngamide K reported by Cao [66]

In 2011, Cao group reported the total synthesis of malyngamides K (Scheme 20), L, and 5″-*epi*-C and confirmed absolute configuration of malyngamide L [66]. For the synthesis of malyngamide K, boronic acid part **167** began from 2-cyclohexen-1-one (**165**). Thus, bromination of enone **165** with bromine generated the bromoenone, followed by protection of the carbonyl group with ethylene glycol to afford the ketal **166**. Ketal **166** was easily transformed to boronic acid **167** by treatment with trimethyl borate in the presence of *n*-butyllithium, and subsequent treatment with hydrogen chloride. The other part amides **172** and **174** began with ethyl propiolate **168**, which was converted to ester **169** in the presence of *n*-tetrabutylammonium iodide. Then reduction of ester gave the intermediate alcohol **170** as a mixture of E- and Z-isomers (E:Z = 1.1:1). The E-isomer could be converted to the desired Z-isomer by irradiation with UV light (> 350 nm) in DCM. Thus, configuration of *Z*-**170** was consistent with that in natural malyngamides K, L, and *epi*-C. Then protection of the hydroxyl group with *p*-toluenesulfonyl group afforded the tosylate **171**, which was transformed into the intermediates **172** and **174**, respectively. The synthesis of amide **172** was also achieved by treatment of tosylate **171** with an excess of aqueous methylamine and followed by amidation with acid **65**. Azidation of **171** and reduction of the obtained azide **173**, followed condensation with the acid **65** afforded **174**. Then malyngamides K was achieved by the Suzuki coupling reaction of **174** and **167**.

**Scheme 21:**
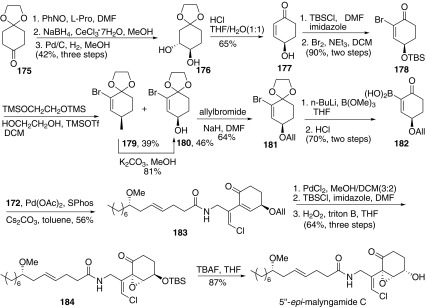
Synthesis of 5″-*epi*-malyngamide **C** reported by Cao [66]

Then the authors completed the more complex malyngamides L, and 5″-*epi*-C (Scheme 21) [66]. For the synthesis of malyngamides 5″-*epi*-C, the key was the synthesis of boronic acid part **182**. Oxidation of **175** using nitrosobenzene gave the corresponding hydroxylamine, which was reduced to afford a single alcohol under Luche conditions, followed by reductive cleavage of the N-O bond to afford diol **176**. Then deketalization and elimination of diol **176** with hydrogen chloride in THF/water (1:1) gave enone **177**. Protection of the hydroxyl group of **177** with *tert*-butyldimethylsilyl chloride and followed by bromination of the corresponding silyl ether gave bromoenone **178**. Then ketalization of ketone **178** gave **179** and **180**. Deprotection of ketal **179** also afforded ketal **180**. Then protection of the hydroxyl group of **180** with allyl bromide afforded the allyl ether **181**. Then the boronic acid **182** was prepared by a procedure similar to that for the preparation of boronic acid **167**. Thus, the skeleton of 5″-*epi*-malyngamide C could be constructed via Suzuki cross-coupling reaction with **182** and previous prepared **172**. Then the allyl ether was converted to silyl ether, followed by stereoselective epoxidation of silyl ether with hydrogen peroxide and benzyltrimethylammonium hydroxide corresponding epoxide **184**. Finally, removal of the TBS protecting group with tetrabutylammonium fluoride (TBAF) provided the 5″-*epi*-malyngamide C, which would be convert to malyngamide C via the Mitsunobu reaction [42].

**Scheme 22:**
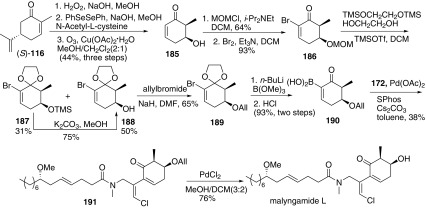
Synthesis of malyngamide **L** reported by Cao [66]

Then malyngamide L was prepared via the similar methodology (Scheme 22) [66]. The authors initially began with (*R*)-(−)-carvone, which finally provided 3″,4″-*epi*-malyngamide K. Therefore, (*S*)-(−)-carvone was chosen as the starting material instead. The preparation of the enone **185** underwent a similar sequence showed in Scheme 18 in the preparation of malyngamide W. Then protection of the hydroxyl group of **185** with MOMCl gave the corresponding ether, followed by bromination to afford the bromoenone **186**. Then boronic acid part **190** was prepared through the sequence as the preparation of boronic acid **182**. Then Suzuki cross-coupling reaction with **190** and **172** afforded **191**, which was removed allyl protecting group to finish malyngamide L.

## Conclusion

This review illustrated a series of lipids which resemble anandamide in structure. At present, only several marine cyanobacterial fatty acid amides have been reported with binding affinities to the cannabinoid receptors, which were grenadamide, mooreamide, semiplenamides A, B, and G, serinolamides A, B and malyngamide B. Others, due to absence of functional assays test, only have the possibility to interact with CB_1_ and CB_2_. Additionally, the metabolites act as receptor agonists implying that they can mediate certain physiological effects through this pathway, which would open more research avenues. Further, a number of total synthesis and well-established synthetic routes have been available; these can assist structural optimization efforts towards more potent analogues, which would be of benefit for understanding the pharmacological mechanisms of cannabinoids and their receptors.

## Abbreviations

*m*-CPBA: 3-Chloroperbenzoic acid
DCC: Dicyclohexylcarbodiimide
DDQ: 2, 3-Dicyano-5,6-dichlorobenzoquinone
PMB: *p*-Methoxybenzyl
IBX: 2-Iodoxybenzoic acid
TBDPS: *tert*-Butyldiphenylsilyl
TBAF: Tetrabutylammonium fluoride
TFA: Trifluoroacetic acid
MOMCl: Chloromethyl methyl ether
HMPA: Hexamethylphosphoramide
PDC: Pyridinium dichlorochromate
EDA: 1-Ethyl-3-(3-dimethylaminopropy)carbodiimide
